# Burden of malaria in Ethiopia, 2000-2016: findings from the Global Health Estimates 2016

**DOI:** 10.1186/s40794-019-0090-z

**Published:** 2019-07-12

**Authors:** Tadele Girum, Teha Shumbej, Misgun Shewangizaw

**Affiliations:** 1 Department of Public health, College of Medicine and Health Sciences, Wolkite University, Wolkite, Ethiopia; 2 Department of Medical laboratory science, College of Medicine and Health Sciences, Wolkite University, Wolkite, Ethiopia; 3 Department of Public health, College of Medicine and Health Sciences, Arba Minch University, Arba Minch City, Ethiopia

**Keywords:** Malaria burden, Mortality, DALY

## Abstract

**Background:**

Globally malaria remains one of the high burden diseases particularly in developing countries. Ethiopia is one of the sub-Saharan countries highly endemic to malaria. Although, recently the burden of malaria was reduced remarkably through public health interventions designed during the Millennium Development Goals, it is still a major public health problem in Ethiopia. Hence, measuring the burden of the disease and assessing the trend is very important for monitoring the extent and changes over a period of time.

**Objective:**

This study aimed to assess the burden of malaria in terms of death and Disability-Adjusted Life Years lost (DALY) between 2000 and 2016.

**Methods:**

The research used data from Global Health Estimate 2016; that originally collected the information through vital registration, verbal autopsy, surveys, reports, published scientific articles, Global Burden of Disease study (GBD 2016) and modeling.

**Results:**

In 2016 there were an estimated 2,927,266 (95% CI, 525,000-6,983,000) new malaria cases in Ethiopia. It caused an estimated 4,782 deaths (95% CI 122.5–12,750) with a crude death rate of 4.7/100,000 and Age-standardized death rate (ASDR) of 4.9/100,000 population. However, the number of deaths due to malaria declined by 54% from the 2000’s record of 10,412 deaths (95% CI 98.8–16180) within 16 years and ASDR declined by 63% from the 2000 record. In the same year, DALY due to malaria was 365,900 years (187,000 years among male and 178,900 years among females). It contributed for 0.78% of the total DALY in Ethiopia and 1% of the global DALY due to malaria. Around 332,100 life years (YLL) were lost and 35,200 years were lived with disability (YLD) due to malaria. Mortality and DALY related to malaria is slightly higher among males; and under 5 children are highly affected.

**Conclusion and recommendation:**

Although, the burden of malaria is remarkably declining in Ethiopia; with a higher level of mortality and DALY, it still remained one of the public health problems. Therefore, strengthening the existing malaria prevention program is important to eliminate the disease within the target period.

## Introduction

Malaria is a global public health problem that causes massive morbidity and mortality and poses a higher burden of disease. It is caused by *Plasmodium* parasites [1]. *Plasmodium falciparum* and *Plasmodium vivax* are the most widely distributed type and pose the greatest public health threat [2]. After sucessful declines were recorded for two decades, malaria started to rise again in the last 2 years. Globally in 2017, there were an estimated 219 million malaria cases and 435,000 deaths. The cases were raised by 2 million from the 2016 report, whereas mortality declined during the same period [3]. Nearly 80% of all mortalities due to malaria occurred in 17 counties, most of them are in Africa and 53% of the death was in 7 countries all in Africa except India [3].

Most malaria cases (200 million or 92%) in 2017 were in the World Health Organization (WHO) African region. The sub-Saharan Africa region was the most affected area contributing for higher share of malaria cases and deaths [3]. In Ethiopia, where three quarters of its territory is considered endemic for malaria putting more than 60 million (60% of the total population) people at risk for infection [4]. Approximately, 4–5 million cases of malaria and 70,000 related deaths have been reported annually in the previous years [4]. Malaria accounted for 30% of the overall DALYs lost [5] and making it a significant impediment to social and economic development.

However, recently the burden of malaria was reduced remarkably in Ethiopia through public health interventions designed during the Millennium Development Goal including early diagnosis and treatment of cases, using artemisinin-based combination therapy (ACT), prevention and control of malaria among pregnant women using intermittent preventive therapy (IPT), use of vector control methods including insecticide-treated bed nets (ITNs), and indoor residual spray (IRS) [6, 7]. As a result, malaria related deaths and admissions in children under the age of five fell by 81 and 73% respectively between 2006 and 2011. Similarly, death and DALY reduced by 94.8 and 91.7% respectively between 1990 and 2015 [7–9].

Despite major progresses have been made to improve the health status of the population through reducing the burden of malaria; it is still a major health problem in Ethiopia. It is among the 10 top leading causes of morbidity and mortality in children under the age of five and adults. Malaria is also ranked at the top of hospital based admissions, outpatient visit and mortality. This may result in failure of malaria elimination goal designed to achieve the sustainable development goal [10]. Although, measuring the burden of malaria is very important to improve the health status of the community there is shortage of recent information. Therefore, this study aimed to measure the burden of malaria in Ethiopia between 2000 and 16 by using Evidence from Global Health Estimate 2018 report (https://www.who.int/healthinfo/global_burden_disease/en/), which will contribute to improve the health status of the population.

## Methods and material

### Study design, settings and population

The burden of disease and cause of mortality was measured using Global Burden of Disease study 2016 approach using Global Health Estimate 2016. The data from 1990 to 2016 for GBD and from 2000 to 2016 for GHE is archived in Institute for Health Metrics and Evaluation (IME) and WHO databases which are freely available for research purpose. This research only measures the burden of malaria in Ethiopia. Ethiopia is the second most populous country in Africa next to Nigeria, with a population estimated at 102 million in 2017 of which 83.86% live in rural areas [11].

### Study variables, sources of data and data collection procedure

The major sources of data for this research is particularly WHO Global Health Estimate database (https://www.who.int/healthinfo/global_burden_disease/en/), which is a compiled data from original estimates conducted by United Nations specialized agencies such as World Health Organization (WHO), World Bank and United Nations Development Program (UNDP). Estimates are available for years 2000, 2005, 2010, 2015 and 2016 for member states and for selected regional groupings of countries, areas and territories.

In addition to this, the GHE used the GBD data as one source of data for its modeling. Institute for Health Metrics and Evaluation (IHME) owns the Global burden of disease study (GBD) and it is available on their database http://www.healthdata.org. The methods used to measure mortality and morbidity is the same in GBD and GHE. However, they are a bit different in the classification of diseases, the source of data and the modeling techniques.

WHO in collaboration with UN partner agencies collect and compile Global Health Statistics and estimates causes of death, population demography and causes of illness through vital registration (VR) data and scientific estimations. In GHE the burden of disease and cause of mortality for the case of Ethiopia was measured using global burden of disease study 2016 approach through surveys and model estimates. This study used the GHE as source of information for population structure and total mortality and then estimated for DALY.

### Operational definition

In this research the following measures of disease burden were defined as the source data from GHE databases and the same classification was used.

- **Disability**: is used broadly in disease burden analyses to refer to departures from good or ideal health in any of the important domains of health
- **Life expectancy**: Average number of years a person from a specific cohort is projected to live from a given point in time.
- **Years of potential life lost (YPLL):** Years of life lost before some arbitrary age (often age 65 or 75). It is Life expectancy minus age at death
- **Disability-adjusted life year (DALY):** is a summary measure which combines time lost through premature death and time lived in states of less than optimal health, loosely referred to as “disability”.

### Statistical analysis and interpretation

The GBD study and GHE approaches to estimate all-cause and cause-specific mortality rates by age, sex and year has been described elsewhere [12–15]. Causes of death by age, sex, and year for all causes and malaria were measured mainly using cause of death ensemble modeling (CODEm) [16]. The model tests a wide range of models, such as mixed effects linear models and spatiotemporal Gaussian process regression (ST-GPR) models, and constructs an ensemble model based on the performance of the different models.

DALY, due to malaria, was measured by summing years of life lost (YLL) due to premature mortality and years lived with disability (YLD), a measure of non-fatal health loss, in a single metric. YLL were estimated using standard GBD methods whereby each death is multiplied by the normative standard life expectancy at each age. YLD were estimated using sequelae prevalence and disability weights derived from population-based surveys. For most sequelae, the GBD 2016 study used a Bayesian meta-regression method, DisMod-MR 2.1, designed to address key limitations in descriptive epidemiological data, including missing data, inconsistency, and large methodological variation between data sources [12–14].

## Results

**Fig. 1:**
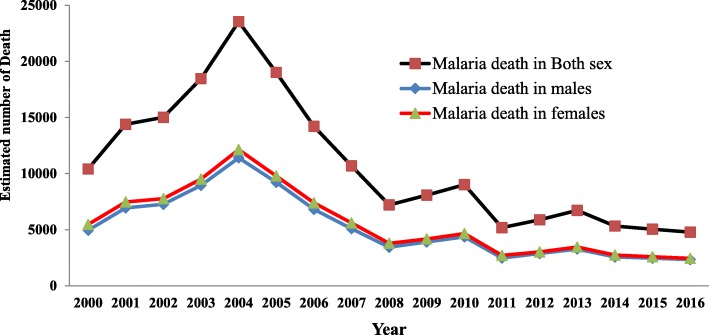
Estimated number of malaria related deaths by sex in Ethiopia, 2000–16

In 2016, there were an estimated 2,927,266 (95% CI: 525,000-6,983,000) new malaria cases in Ethiopia. Despite the population at risk were increased from 59,637,819 to 69,634,176 between 2010 and 2016, the number of cases declined by 60%. In the same year, malaria caused an estimated 4,782 deaths (95% CI 122.5–12,750). It is estimated to cause a crude death rate of 4.7/100,000 and ASDR of 4.9/100,000 population. However, the number of deaths due to malaria was declined by 54% from the 2000’s record of 10,412 deaths (95% CI 98.8–16,180) within 16 years and ASDR declined by 63% from the 2000 record (Tables 1, 2 & Fig. 1).

**Table 1:** Estimated number of malaria related death (in thousands) by causes, year and age in Ethiopia, 2000–16

		Year				Age						
Gender	Cause/disease	2000	2010	2015	2016	0-4	5-14	15-29	30-49	50-59	60-69	70+
Ethiopia												
Both	All Causes	982	738.4	710.6	700.1	187.1	49.3	74	99.9	50.3	71.7	167.9
Both	Infectious and parasitic	405.4	218.4	184	168.7	38.7	25.7	24.4	31.6	11.6	12.1	24.5
Both	Malaria	10.4	9	5	4.8	1.3	1.2	0.7	0.6	0.2	0.3	0.4
Male	All Causes	528.6	397.1	382.7	378	103.5	27.8	44.8	54.4	27.2	37.8	82.2
Male	Infectious and parasitic	217.3	115.6	97.6	90.6	20.1	13.7	14.4	17.5	6.5	6.4	12
Male	Malaria	5.5	4.7	2.6	2.4	0.7	0.6	0.4	0.3	0.1	0.15	0.2
Female	All Causes	453.4	341.3	328.1	322.1	83.6	21.5	29.2	45.5	23.1	33.9	85.3
Female	Infectious and parasitic	188.1	102.8	86.1	78	18.6	12	10	14.2	5.2	5.7	12.5
Female	Malaria	4.9	4.4	2.5	2.3	0.8	0.6	0.3	0.3	0.1	0.15	0.2
Africa												
Both	All Causes	9729.6	9029.6	8843.2	8845.1							
Both	Infectious and parasitic	4318	3314	2822.7	2728.7							
Both	Malaria	706.8	527.63	419	408.12							
Global												
Both	All Causes	52307.4	54124.7	56271.8	56873.8							
Both	Infectious and parasitic	8550.3	6564.2	5651.4	5491.4							
Both	Malaria	767.33	580.6	455.4	446.45

**Table 2:** Crude death rate (CDR) and age standardized death rates (ASDR) per 100,000 populations in Ethiopia, 2000–2016

		Year			
Gender	Cause of death	2000	2010	2015	2016
CDR/100,000					
Both	All Causes	1475.8	841.9	711.7	683.7
Both	Infectious & parasitic diseases	609.3	249	184.3	164.7
Both	Malaria	15.6	10.3	5.1	4.7
Male	All Causes	1593	907.3	767.6	739.3
Male	Infectious & parasitic diseases	654.9	264.1	195.8	177.3
Male	Malaria	16.5	10.6	5.2	4.8
Female	All Causes	1359	776.8	656	628.2
Female	Infectious & parasitic diseases	563.9	233.9	172.7	152.2
Female	Malaria	14.8	9.9	4.9	4.6
ASDR/100,000					
Both	All Causes	1816.7	1213.6	1074	1048.3
Both	Infectious & parasitic diseases	688.5	320.6	236.3	218.9
Both	Malaria	13.3	9.8	5.3	4.9
Male	All Causes	1959	1323.1	1177.5	1152.9
Male	Infectious & parasitic diseases	751	348.6	257.4	241.3
Male	Malaria	14.6	10.4	5.6	5.2
Female	All Causes	1678.1	1111.8	978.8	952.1
Female	Infectious & parasitic diseases	627.8	294.6	216.9	198.3
Female	Malaria	12.1	9.2	5.1	4.7

Malaria related mortality in Ethiopia have contributed for 2.8% (4,782/168,700) of infectious and parasitic disease mortality and 0.7% (4,782/700,100) of all deaths by the year 2016. Similarly, malaria mortality in Ethiopia has contributed for 1.2% (4,782/408,125) of malaria related mortality in Africa and 1.07% (4,782/446,446) of global malaria mortality. The percentage share of malaria for the total mortalities recorded in Ethiopia, Africa and globally in general is declining throughout the periods between 2000 and 2016.

Mortality due to malaria was highest among males and under five children. Of the 4,782 malaria related mortalities, more than 2,400 deaths were among males. The ASDR was 5.2/100,000 population among males and 4.7/100,000 population among women. Similarly, crude death rate due to malaria was 4.8/100,000 population in males and 4.6/100,000 population in females. Also the highest malaria related mortality of 1,300 deaths was recorded among children under the age of five and 1,200 deaths were recorded among children aged 5–14 years.

In 2016, Years Lived with Disability (YLD) due to malaria was 33,800 years (17,500 years for males and 16,300 years for females) and no difference was observed since 2000. In the same year, around 332,100 life years (YLL) were lost due to malaria related premature mortality. Thus, malaria contributes for 0.8% (332,100/37,840,800) of the total potential life years lost due to premature deaths. Also, the YLL due to malaria is continuously declining in Ethiopia (Table 3).

**Table 3:** Disability adjusted life years (DALY) in thousands in Ethiopia, 2000–2016

		Year			
Gender	Cause of DALY	2000	2010	2015	2016
DALY in thousands					
Both	All Causes	71354	51371.4	48026.1	46507.4
Both	Infectious & parasitic diseases	28666.6	14684.4	12378.2	11042.7
Both	Malaria	839.1	695.1	385.6	365.9
Male	All Causes	38313	27697.9	26008.9	25282.4
Male	Infectious & parasitic diseases	15308.9	7747.2	6562.7	5938.2
Male	Malaria	433.6	355.9	197	187
Female	All Causes	33041	23673.5	22017.1	21225
Female	Infectious & parasitic diseases	13357.7	6937.2	5815.5	5104.5
Female	Malaria	405.5	339.1	188.6	178.9
YLD in thousands					
Both	All Causes	5978.3	7535.	78527.4	8666.6
Both	Infectious & parasitic diseases	803.	909.1	906	4911.7
Both	Malaria	35.2	30.1	33.7	33.8
YLL in thousands					
Both	All Causes	65375.7	43835.7	39498.7	37840.8
Both	Infectious & parasitic diseases	27863.2	13775.3	11472.2	10130.9
Both	Malaria	803.8	665	351.9	332.1

Meanwhile, DALY due to malaria was 365,900 years (187,000 years among male and 178,900 years among females) in Ethiopia by the year 2016. By the same year, it contributed for 0.78% of the total DALY due to all causes in Ethiopia and 1% of the global DALY due to malaria. DALY lost due to malaria was highest among children under the age of five; where 132,600/365,900 (36.3%) of the total malaria related disability adjusted life years were recorded. As it was in the mortality, DALY due to malaria shows a declining trend between 2000 and 2016 at the national and international levels (Table 3).

## Discussion

This study assessed the burden of malaria in Ethiopia from 2000 to 2016 evidenced from the GHE 2016 (reported in 2018). The burden was measured in terms of morbidity, mortality, years lived with disability, years of potential life lost and disability adjusted life years. The trends over time, gender differences and age difference were measured and its contribution for the global malaria burden was also computed. It is found that the burden of malaria particularly; malaria related mortality rate and disability adjusted life years lost due to malaria is declining related to interventions taken at the millennium development goal.

Despite the population at risk was increased by 16.75% between 2010 and 2016, estimated numbers of new malaria cases declined by 60% [3]. Programs implemented to achieve the Millennium Development Goal including insecticide treated bed net (ITN) distribution, drainage of stagnant water, indoor residual spray (IRS), improved health care seeking behavior for fever, prevention and control of malaria among pregnant women by using intermittent preventive therapy (IPT), and improved accessibility to Artemisinin-based combination therapy (ACT) may have remarkably contributed for these achievement [3, 6, 17].

Accordingly, the number of deaths due to malaria declined by 54% within 16 years from the 2000’s record of 10,412 deaths (95% CI 98.8–16,180) to 4,782 deaths (95% CI 122.5–12,750) in 2016. In the same year, crude death rate (CDR) and ASDR declined by 70 and 63% respectively. It was also evident from the WHO and Ministry of Health report that, malaria incidence and related mortality has been declined by 50–75% between 2000 and 2013. Similarly, malaria incidence and mortality rates due to *Plasmodium falciparum* have declined by more than 50% between 2010 and 2015. Thus, Ethiopia has achieved the Millennium Development Goal targeted to halve mortality rate from malaria [3, 6, 9, 10].

With strong government leadership, the implementation of primary healthcare program and effective implementation of the malaria control strategies at grassroot level has led Ethiopia to reduce the burden of malaria faster than in most of Sub-Sahara African countries [18]. However, Ethiopia still have high burden of malaria which accounts for 6% of global malaria cases and 12% of the global cases and deaths due to *Plasmodium vivax*. Hence, Ethiopia is one of four countries that carry more than 75% of deaths and cases due to *P. vivax* [3, 18]*.*

In 2016, YLD, YLL and DALY due to malaria was 33,800 years (17,500 years for males and 16,300 years for females), 332,100 years (169,600 years for males and 162,500 years for females) and 365,900 years (187,000 years among male and 178,900 years among females) respectively. Indicating that, DALY from malaria has contributed for 0.78% of the total DALY in Ethiopia and 1% of the global DALY due to malaria. In most African countries malaria is the major cause of mortality and morbidity. The role of malaria related DALY in these countries was higher than what has been reported in Ethiopia [3, 18].

The national malaria prevention and treatment programs have made considerable progress in addressing the epidemic and averted many more new infection and malaria related death. Since then, the burden of malaria infection had declined at the national and regional levels through different public health interventions. However, still malaria is a public health problem in the country with higher rate of morbidity and mortality particularly among children under the age of five [1, 4, 5]. Hence, Ethiopia may be challenged to achieve the sustainable development goal related to malaria elimination and global technical strategy (GTS) for malaria eradication program designed to reduce malaria by 90% [19].

The findings of this study might suffer from the fact that it is secondary data based on records; the reliability of the recorded data couldn’t be ascertained and potential bias associated with estimation is there. Some methodological problems may have encountered in this research. Most of the data was originally estimated from model predictions and data source for the model was either reports of vital registration or sample survey that could again affect the reliability of the data. Moreover, the forecasted values from the trend may change through time due to change in intervention programs; this in turn affect the reliability of the estimate.

## Conclusion and recommendation

The burden of malaria is remarkably declining in the last two decades in Ethiopia. However, with a higher level of mortality and DALY, malaria still remained one of the public health problems. Therefore, malaria control and elimination strategies should be strengthened to further reduce the incidence and burden of malaria particularly among highly affected age groups during the implementation periods of sustainable development goal (SDG) and malaria elimination program that are undertaking by the government.

## Data Availability

The GBD 2015 data is available at the GBD website (https://vizhub.healthdata.org/gbd-compare/) and Global Health Estimate 2018 is also available at: (https://www.who.int/healthinfo/global_burden_disease/en/) both are freely accessible.

## Abbreviations

ACT: Artemisinin-combination therapy
CODEm: Causes of death ensemble modelling
DALY: Disability-adjusted life years lost
GBD: Global burden of diseases
GTS: Global technical strategy for malaria
IPT: Intermittent preventive therapy
IRS: Indoor residual spray
ITN: Insecticide-treated bed nets
MDG: Millennium development goals
SDG: Sustainable development goals
WHO: World Health Organization
YLD: Years of lived with disability
YLL: Years of life lost due to mortality
